# Effectiveness of Radiofrequency in Treating Pain Associated With Trigeminal Neuralgia in Oncologic Patients

**DOI:** 10.7759/cureus.76965

**Published:** 2025-01-05

**Authors:** Tania H Ahuactzin Avendaño, Rocio Guillen, Karen Patricia Segovia Sandoval, Angel Juarez, Ana Lady Sanchez Ortega, Frida Paola Viveros Aguilar

**Affiliations:** 1 Pain Medicine, National Cancer Institute, Mexico City, MEX; 2 Pain Medicine, Clinica Alive, Mexico City, MEX; 3 Pain Management, National Cancer Institute, Mexico City, MEX

**Keywords:** cancer, facial pain, pain management in oncologic patients, radiofrequency, trigeminal nerve neuralgia

## Abstract

Introduction

In cancer patients, trigeminal neuralgia (TN) has been not only observed in its secondary form but also described as one of the common neuropathic pain syndromes related to cancer. Patients with TN experience a marked reduction in quality of life due to the nature and severity of the pain. Although the standard treatment is microvascular decompression, this approach is not always feasible in oncologic patients. Therefore, radiofrequency (RF) guided by tomography has been described as an ablative intervention that allows achieving immediate pain relief in TN. It is of interest to identify the therapeutic response to pain in oncologic patients treated by this procedure.

Objective

The study aimed to evaluate the effectiveness of percutaneously guided tomography-assisted RF in oncologic patients with TN.

Materials and methods

We conducted an observational, retrospective, longitudinal, and analytical study. Patients with cancer and TN treated at the Pain Clinic of the National Cancer Institute in Mexico, who underwent RF, were included. A non-probabilistic sampling of consecutive cases was used, and selection criteria were applied. Clinical records were reviewed to measure study variables, including pain by TN before and after the procedure, as well as patient satisfaction. Data were analyzed using descriptive and inferential statistics, employing Student's t-test for related samples, with a significant level set at p ≤ 0.05 to determine statistical significance. The recorded data were analyzed using IBM SPSS Statistics for Windows, V. 26.0 (IBM Corp., Armonk, NY, USA).

Results

In the sample of 76 subjects (50% women and 50% men) with a mean age of 60.6 years, a variety of oncologic diagnoses were presented, with skin and soft tissue tumors being the most frequent, mainly located at the level of the head and neck. The etiology of TN was secondary in most cases (89.5%), with similar anatomical involvement on both hemifaces (50% left, 50% right). The branches most frequently involved were the maxillary nerve (V2) and mandibular nerve (V3) (39.5%) and V3 alone (22.4%). The most common probable cause was tumor activity (53, 69.7%). Most patients were receiving anticonvulsant treatment (carbamazepine-oxcarbazepine) (58.9%), followed by gabapentinoids (31.5%). Of the 76 patients, only six received computed tomography (CT)-guided RF. No complications associated with RF application were reported. The mean baseline pre-procedure pain (3.8 ± 2.48) and mean baseline post-procedure pain (2.17 ± 1.94) were significantly different in the Wilcoxon signed-rank test (p = 0.043), as were the mean incidental pre-procedure pain (9.0 ± 1.09) and mean incidental post-procedure pain (5.8 ± 2.31) (p = 0.019).

Conclusions

Our research provides relevant data on the effectiveness of RF in managing TN in oncologic patients, demonstrating a clinically and statistically significant reduction in pain intensity. These findings need to be confirmed through prospective, longitudinal, long-term studies with control groups in a larger number of patients.

## Introduction

According to the International Association for the Study of Pain (IASP), trigeminal neuralgia (TN) is classically characterized by recurrent, evocative, unilateral, brief, shock-like pains with abrupt onset and cessation, affecting one or more divisions of the trigeminal nerve [1]. It occurs most frequently in women over 50 years old, is almost always unilateral, and mainly affects the second and third branches of the trigeminal nerve. Currently, the treatment of TN is divided into pharmacological and surgical procedures. Neurosurgical treatment consists of major procedures including microvascular decompression and minimally invasive percutaneous treatments, among them radiofrequency (RF) application. RF is an effective treatment strategy for TN due to its excellent rates of therapeutic success [2].

The fundamental principle of RF treatment for TN is the blockade of pain signal conduction through the application of high temperatures, this achieving modulation of the nociceptive function of the nerve. Elderly patients of those with comorbidities, such as cancer patients, benefit from the minimally invasive nature and safety profile offered by RF [3].

The earliest records of TN date back to the first century in the writings of Galen, Aretaeus of Cappadocia, and Avicenna, although the first official descriptions were not documented until the 18th century. In 1756, Nicholas André coined the term "tic douloureux" due to the characteristic facial spasms that accompany pain episodes. The English physician John Fothergill is credited with being the first to give a complete and accurate exposition of the disorder in a presentation to the Medical Society of London in 1773, titled "On a painful affliction of the face." Hence, this condition is also known as "Fothergill's disease" [4].

The International Classification of Headache Disorders, in its third edition (ICHD-3), defines TN as unilateral, brief, electric shock-like pain of sudden onset and cessation, limited to the distribution of one or more branches of the trigeminal nerve division and triggered by innocuous stimuli. It may develop without apparent cause or be caused by another diagnosed disorder. In the latest edition of the ICHD-3, TN is classified into three main types: classical, secondary, and idiopathic [5].

The trigeminal nerve or fifth cranial nerve (V cranial nerve) is a mixed nerve with both motor and sensory functions [6].

It divides into three branches: the ophthalmic nerve or V1, the maxillary nerve or V2, and the mandibular nerve or V3. The most affected branch is the maxillary branch, while the least involved is the ophthalmic branch, and it generally occurs unilaterally.

The pathophysiology that best explains TN and is most widely accepted is the compression of the sensory portion of the trigeminal nerve near the entry zone of the root into the pons by a small adjacent branch of the basilar artery, most commonly the superior cerebellar artery. This may lead to anatomical alterations in the nerve such as atrophy and loss of its myelin sheath [7].

Vascular compression is the cause of demyelination; when the myelin sheath thins enough, it allows the transmembrane passage of ions into the axon, resulting in membrane depolarization and consequently hyperexcitability.

Histological evidence indicates that the nerve fibers involved in this demyelination process are A-β fibers (large non-nociceptive fibers), which are the most susceptible to demyelination due to mechanical damage or multiple sclerosis. It has been proposed that high-frequency discharges originating at the site of demyelination along the primary A-β fibers are redirected by brainstem neurons to be perceived as paroxysmal pain [8].

RF treatment is a viable option with initial and long-term clinical efficacy and reliability, which can improve pain almost instantly in 90-100% of cases [9].

The fundamental principle of RF treatment for TN is to block the conduction of the pain signal by using high temperatures of up to 90°C to destroy the nerve or modulate the nociceptive function of the trigeminal nerve with a temperature not exceeding 42°C. Therefore, by interfering with the function of the trigeminal nerve or destroying the integrity of its anatomical structure, therapeutic effects can be achieved [10].

The percutaneous puncture of the foramen ovale has evolved from fluoroscopic guidance to the use of computed tomography (CT) and, more recently, the employment of various neuro-navigation systems in an attempt to maximize outcome and minimize morbidity and mortality from the inadvertent puncture of vital structures around the foramen ovale [11].

CT image reconstruction for the treatment of TN allows for the precise placement of the RF needle at the opportune moment, not only enhancing the practitioner's ability but also making the patient more comfortable by shortening operative time [11].

Neurolysis is performed at temperatures of 60-80°C for 30-60 seconds [12].

Percutaneous RF is described as a procedure with low morbidity and no mortality. The most frequently reported complication is sensory loss in the treated branch paralysis of the masseter muscle, as well as the presence of cheek hematoma that resolves within a few days. Other less common complications include corneal keratitis, hypoesthesia, and temporary paralysis of the third and fourth cranial nerves [13].

In a systematic review conducted by searching the PubMed, Embase, and Cochrane databases (up to July 31, 2020), the primary reported complication was facial hypoesthesia. Complications involving corneal affection were particularly high when RF was used to treat the ophthalmic division (V1) of TN [14].

TN has an annual incidence of three to five cases per 100,000 people worldwide, with few specific records in Mexico due to its treatment by various medical specialties, including stomatologists [15]. A retrospective study at the General Hospital of Mexico (1990-1999) found a frequency of 3.8 cases per 1,000 patients, primarily affecting women, with an average age of onset, and predominantly on the right side of the face, mainly involving the second branch of the trigeminal nerve [16]. A similar study at the "Dr. Manuel Gea González" Hospital (2009-2019) also observed a higher prevalence in women (62.3%), with an average age of 60.2 years and primarily affecting the left side of the face [17].

Both studies confirm international epidemiological patterns, showing that TN primarily affects women over 50 years old, in a unilateral form, and involves the second and third branches of the trigeminal nerve [18]. In cancer patients, TN has been observed not only as a secondary condition but also as a common neuropathic pain syndrome caused by tumor activity [19]. Tumors in the nasopharynx, lymphoma infiltrations, and metastases from melanoma, breast cancer, colon cancer, and prostate cancer have been described as oncological causes. Post-oncological treatment of TN (due to radiation therapy) has also been documented [20].

Patients with TN experience a significant reduction in their quality of life due to the severity of the pain, often leading to psychological distress, including suicide attempts. The incidence of depression and anxiety in these patients is nearly three times higher than in the general population and positively correlates with pain levels and disease duration. Mood disorders improve when pain is relieved, resulting in a better quality of life [21].

## Materials and methods

We conducted an observational, retrospective, longitudinal, and analytical study at the Pain Clinic of the National Cancer Institute in Mexico. The study focused on patients with cancer and TN who underwent RF treatment between January 1, 2018, and December 31, 2022. Our project, registered under number 2023/124, received authorization from the Research Committee of the National Cancer Institute, as it involved a risk-free examination of archived clinical records, obviating the need for informed consent. A non-probabilistic sampling of consecutive cases was used, and selection criteria were applied. Clinical records were reviewed to measure study variables, including pain by TN before and after the procedure, as well as patient satisfaction. Data were analyzed using descriptive and inferential statistics, employing Student's t-test for related samples, with a significant level set at p ≤ 0.05 to determine statistical significance. The recorded data were analyzed using IBM SPSS Statistics for Windows, V. 26.0 (IBM Corp., Armonk, NY, USA).

## Results

A total of 89 patients were included, of which 13 were excluded during the study for not meeting the corresponding inclusion criteria (Table 1), forming a sample of 76 patients diagnosed with TN, of whom six received interventional treatment with RF application guided by CT. Half of the patients were women (n = 38.50%) and the other half were men (n = 38.50%). The age ranged from 21 to 90 years, with an average of 60.6 years. The weight ranged from 33 to 158 kg with a mean of 62.7 kg, and the height ranged from 1.42 to 1.85 m with a mean of 1.59 m. The BMI ranged from 15.07 to 61.72 kg/m^2^ with a mean of 24.7 kg/m^2^. By BMI categories, we observed underweight (n = 7, 9.2%), normal weight (n = 41, 53.9%), overweight (n = 19, 25%), obesity GI (n = 5, 6.6%), obesity GII (n = 3, 3.9%), and obesity GIII (n = 1, 1.3%).

**Table 1:** Exclusion and inclusion criteria in the study.

Exclusion criteria in the study	Inclusion criteria in the study
Patients without a cancer diagnosis	Men and women diagnosed with cancer who have been treated at the Pain Clinic of the National Cancer Institute in Mexico
Patients with an adequate response to pharmacological treatment	Patients aged 18 years and older
Patients under 18 years of age	Patients with a clinical diagnosis of trigeminal neuralgia (in any of its branches, whether unilaterally or bilaterally)
Patients treated with any interventional procedure that is not the reason for the study (tomography-guided radiofrequency) and who do not have a complete clinical record	Patients who have undergone an interventional procedure with tomography-guided radiofrequency

**Figure 1:**
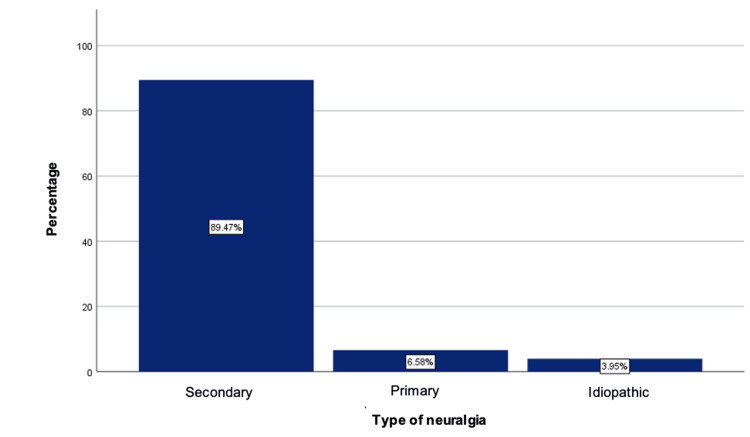
Type of trigeminal neuralgia.

**Figure 2:**
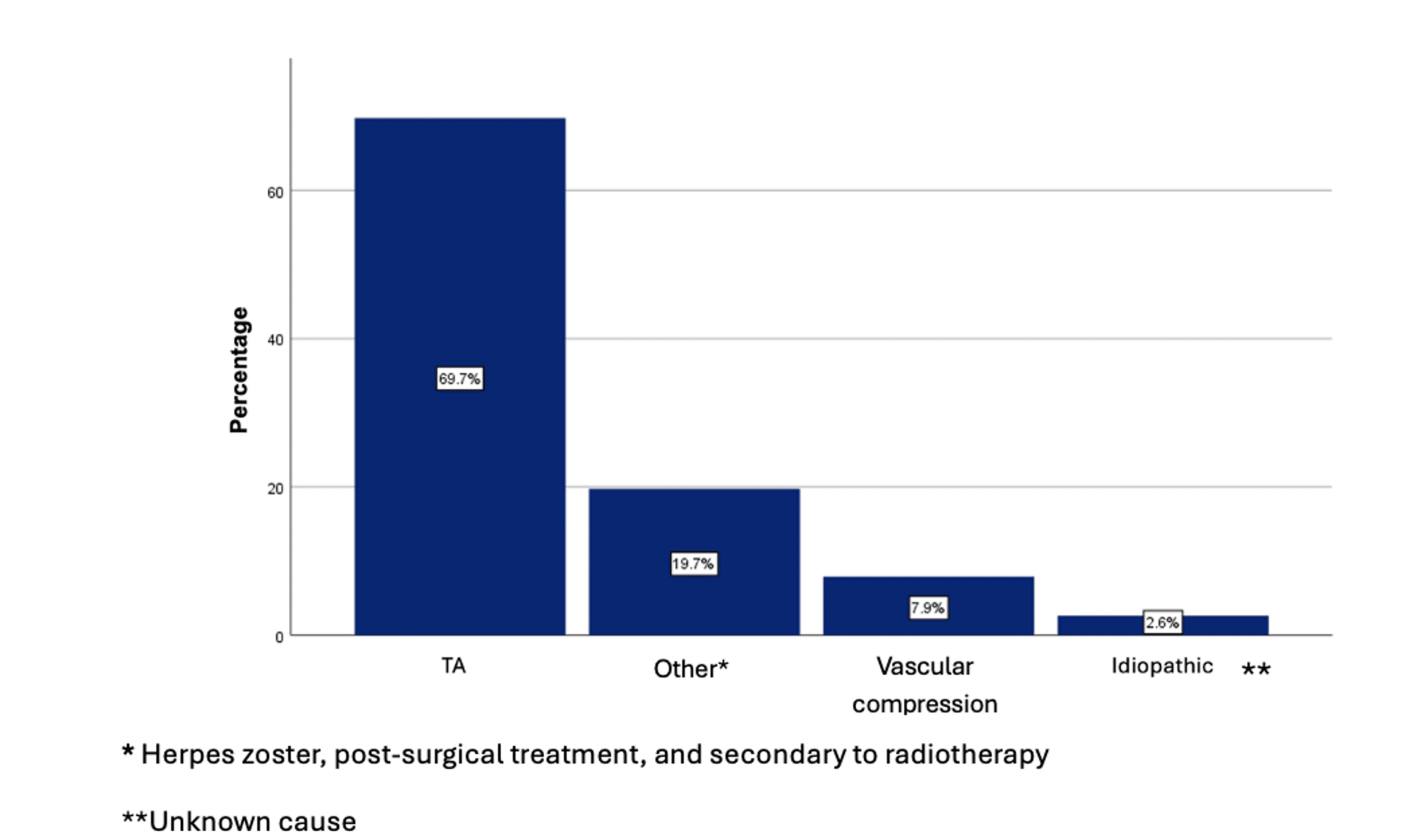
Probable cause of trigeminal neuralgia. TA: tumor activity

Regarding the type of TN, the secondary form was the most common, present in the majority of patients (89.5%), followed by the primary form in five patients (6.6%) and the idiopathic form in three patients (3.9%) (Figure 1). About the secondary form of TN, which is caused by an underlying disorder, the main cause identified in the study was tumor activity (69.7%) (Figure 2).

**Figure 3:**
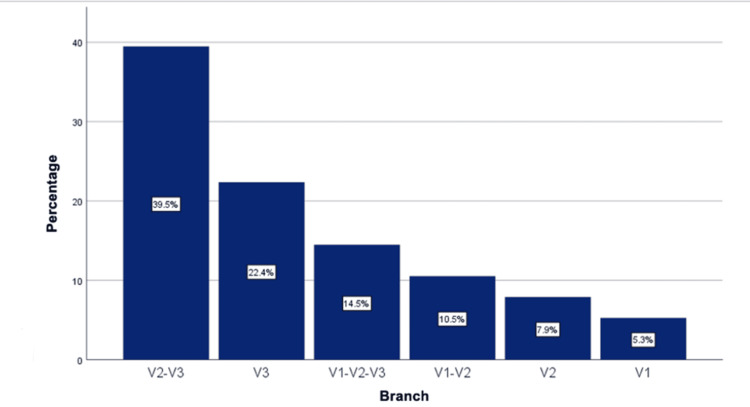
Branches of the trigeminal nerve affected.

The anatomical involvement was similar on both sides of the face (50% left, 50% right). The branches involved were most frequently the maxillary and mandibular branches (V2-V3) in 30 patients (39.5%), secondly the mandibular branch (V3) in 17 patients (22.4%), and thirdly all three branches (V1, V2, V3) in 11 patients (14.5%), in addition to cases involving the ophthalmic and maxillary branches (V1, V2) (n = 8, 10.5%), maxillary branch (V2) (n = 6, 7.9%), and ophthalmic branch (V1) only (n = 4, 5.3%) (Figure 3).

**Figure 4:**
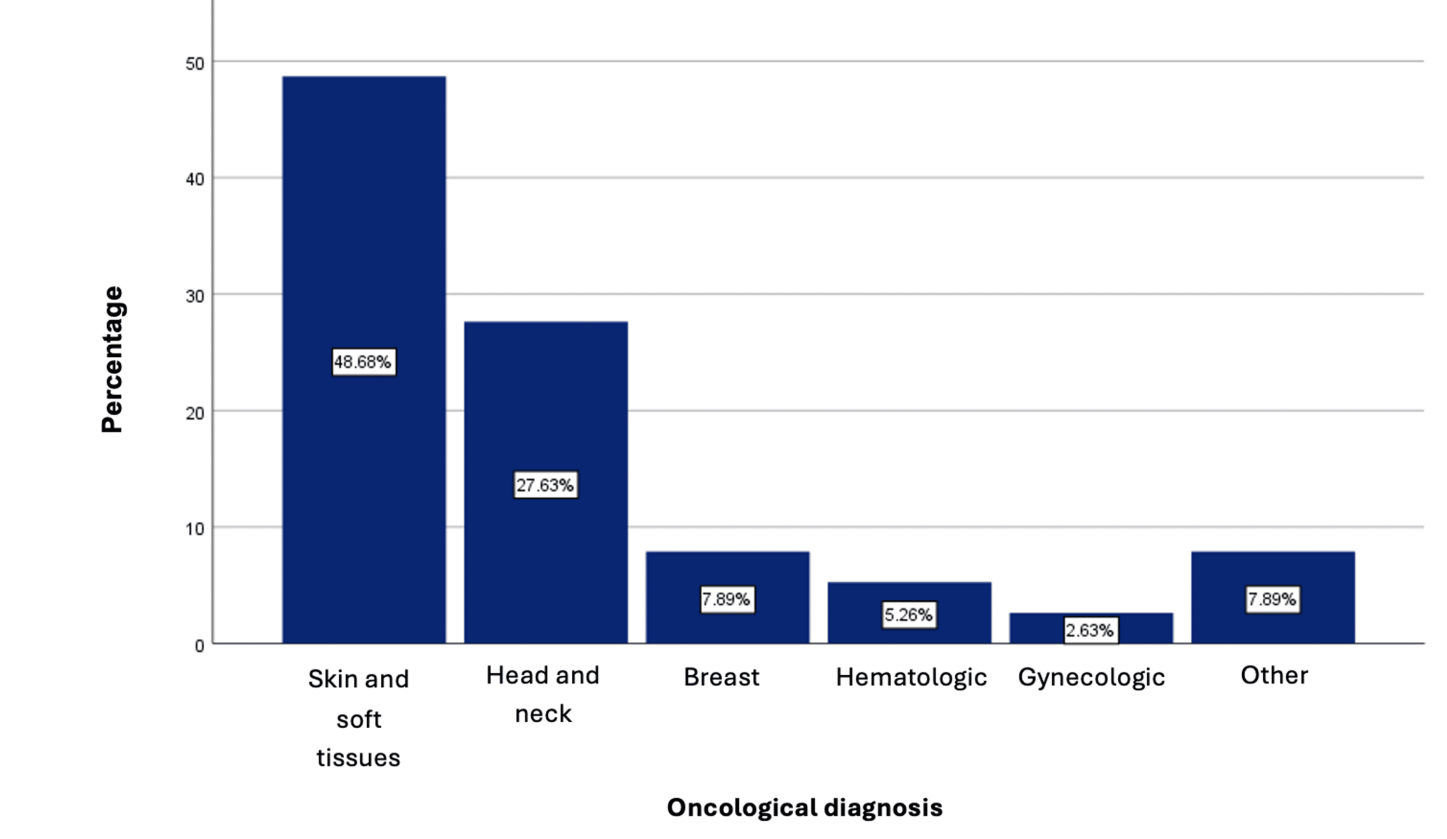
Oncological diagnosis of the patients.

The most common oncological diagnosis was skin and soft tissue tumors (n = 37, 48.7%), with squamous cell carcinoma being the most described type in this group of patients, followed by head and neck tumors (n = 21, 27.6%), breast tumors (n = 6, 7.9%), hematologic tumors (n = 4, 5.3%), gynecological tumors (n = 2, 2.6%), and others (n = 6, 7.9%) (Figure 4).

The treatment of TN consisted mostly of an anticonvulsant (n = 43, 58.9%, carbamazepine or oxcarbazepine), followed by gabapentinoids (n = 23, 31.5%), and, thirdly, tricyclic antidepressants (n = 3, 4.1%) and multimodal treatments (with gabapentinoids plus an anticonvulsant (n = 3, 4.1%) or gabapentinoids plus tricyclic antidepressants (n = 1, 1.4%)). Three patients (3.9%) did not receive a neuromodulator treatment. Regarding opioid therapy, opioids with other mechanisms of action (norepinephrine reuptake inhibition or serotonin and norepinephrine reuptake inhibition) were prescribed in 32 patients (42.1%), pure opioid agonists in 27 patients (35.5%), and partial opioid agonists in 13 patients (17.1%), and four patients (5.3%) did not receive opioid treatment.

Out of the 76 patients, only six received CT-guided RF. The pain before the procedure was baseline from 1 to 7 with an average of 3.8 and a median of 3.5 (ẋ = 3.8; Me = 3.8; s = 2.48) and incidental from 8 to 10 with an average of 9.0 and a median of 9.0 (ẋ = 9.0; Me = 9.0; s = 1.09). All patients were evaluated one week after the procedure, and no complications associated with RF application were reported. Furthermore, post-procedure baseline pain ranged from 0 to 5 with an average of 2.17 and a median of 2.5 (ẋ = 2.17; Me = 2.5; s = 1.94) and incidental from 3 to 9 with an average of 5.8 and a median of 5.5 (ẋ = 5.8; Me = 5.5; s = 2.31) (Table 2).

**Table 2:** Pre- and post-procedure pain evaluation (evaluation one week after the procedure). *p ≤ 0.001 Wilcoxon signed-rank test NRS: numerical rating scale

Variable	Minimum	Maximum	Mean	Median	Standard deviation		Interquartile range	P-value
Baseline pre-procedure pain (NRS)	1	7	3.83	3.50		2.48	4.50	0.043*
Baseline post-procedure pain (NRS)	0	5	2.17	2.50		1.94	3.50	
Incidental pre-procedure pain (NRS)	8	10	9.00	9.00		1.09	2.00	0.019*
Incidental post-procedure pain (NRS)	3	9	5.83	5.50		2.31	4.50	

**Figure 5:**
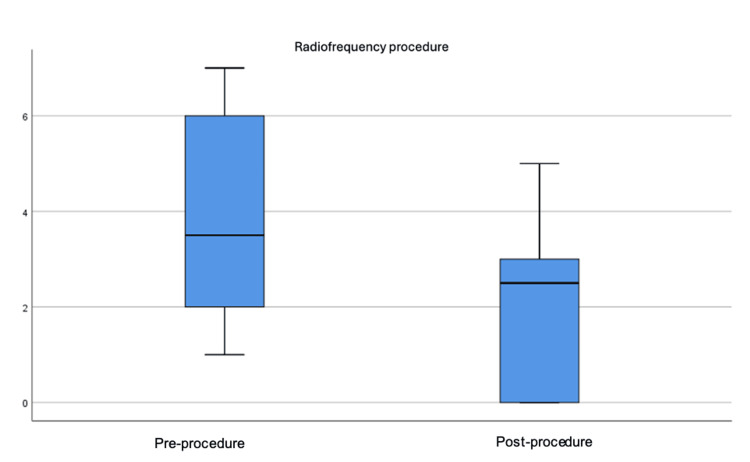
Boxplot of the distribution of baseline pain severity before and after the procedure (p ≤ 0.01).

The differences between the pre-procedure and post-procedure baseline averages were statistically significant using the Wilcoxon signed-rank test (Z = -1.29; p = 0.043); likewise, the differences between pre- and post-procedure incidental averages were significant (Z = -2.02; p = 0.019), with more pronounced differences in incidental pain than baseline (Figure 5).

**Figure 6:**
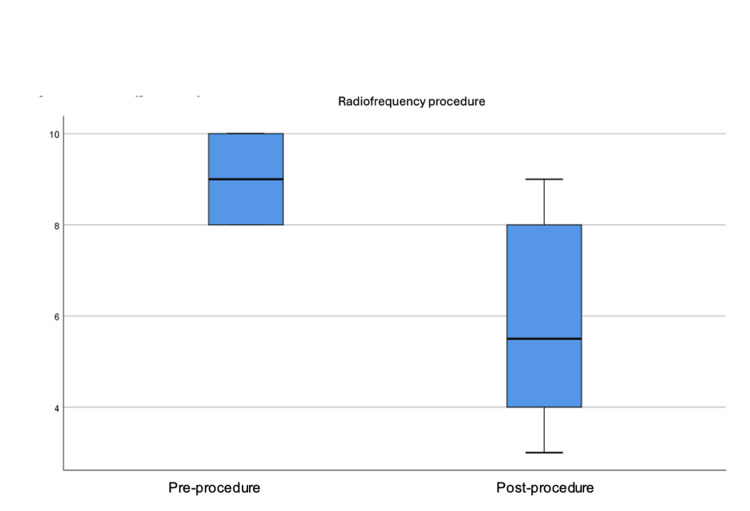
Boxplot of the distribution of incidental pain severity before and after the procedure (p ≤ 0.01).

As observed in the study, pain decreased in all patients (100%) on average one week after the procedure (Figure 6).

Regarding the level of satisfaction described by patients using the Likert scale, three out of six reported being "satisfied" and two "very satisfied".

## Discussion

​​​​​​TN in oncologic patients can be more challenging due to the complexity of its management, which includes limited therapeutic options and the need to alleviate pain in the context of underlying neoplastic disease. Among the therapeutic alternatives, RF has emerged as a promising strategy for pain management, especially in oncologic patients, due to its effectiveness, minimally invasive nature, and safety profile.

The present study evaluated the effectiveness of percutaneous RF guided by tomography in oncologic patients with TN between 2018 and 2022. A total of 76 patients were included, half of whom were women and the other half men, with an average age of 60.6 years. The majority presented with secondary neuralgia (89.5%), with the affected hemiface being indistinct in the sample. The most commonly affected branches were V2-V3, followed by V3, and, to a lesser extent, the three branches V1-V2-V3 and other combinations.

The primary cause of TN was tumor activity in the majority of cases (69.7%), with skin and soft tissue tumors and head and neck tumors being the most frequent oncologic diagnoses. Before RF treatment, patients received various pharmacological therapies, with anticonvulsants and gabapentinoids being the most common. Regarding pain management, the use of opioids in different modalities was frequent.

The study did not record any complications associated with RF application, within the first seven days after the procedure, supporting its safety profile in this clinical context. Although not described in all patients, the majority reported a high level of satisfaction with the therapy, as reflected in the Likert scale. Additionally, pain assessment using the numerical rating scale revealed a statistically significant decrease in post-procedure pain compared to baseline (p = 0.043).

The pain, measured by the verbal rating scale (VRS), was mostly moderate to severe before the procedure, with a marked reduction in the VRS measured at assessment one week after RF treatment. The mean difference between initial and final incidental pain was statistically significant (p = 0.019), demonstrating an overall decrease in 100% of the patients.

The results obtained support the effectiveness of percutaneous RF guided by tomography in treating pain associated with TN in oncologic patients. The statistically significant decrease in post-treatment pain points towards the effectiveness of this therapeutic modality, providing relief to a population with specific and often complex pain management needs.

It is important to note that in our study, while the sample size was 76 patients, only six of them were treated with CT-guided RF application, which weakens the obtained results. However, considering that the number of patients admitted due to cancer and TN was not so small, we were able to identify sociodemographic variables, the pain pattern (baseline + incidental), with incidental pain episodes producing high pain scores despite most study subjects having good control of baseline pain symptoms. We were also able to characterize both the most prevalently affected branches of the trigeminal nerve and the oncological processes that frequently generated this pain entity, along with the therapeutic modalities to counteract it. In this latter point, we observed that pharmacological measures are carried out with anticonvulsants (carbamazepine and oxcarbazepine), which have been classified as the first-line treatment by different authors and international guidelines such as the American Academy of Neurology, the European Academy of Neurology, and the National Institute for Health and Care Excellence, according to the Headache Study Group of the Spanish Society of Neurology and the Royal College of Surgeons of England to mention a few [22-24].

One of the main limitations, aside from the small sample size subjected to RF application, is the lack of detailed information regarding the specific type of RF procedure used in each case. This omission makes it challenging to fully comprehend the techniques applied. Furthermore, the absence of direct comparisons with other therapeutic approaches for TN creates a gap in evaluating the effectiveness of RF in relation to alternative treatment options.

Additionally, the lack of detailed patient satisfaction follow-up and accurate description of satisfaction levels represents a limitation in fully understanding the patient's experience and their subjective perception of the treatment. This can influence the overall interpretation of the results, as patient satisfaction is a crucial component in evaluating therapeutic success.

Although the study demonstrates a statistically significant reduction in pain following RF treatment, the mentioned limitations suggest the need for broader future research. These studies could address the identified deficiencies through a prospective approach, standardizing the procedure, including patient satisfaction assessments, quality-of-life evaluations, and even economic impact assessments, as well as direct comparison with other therapeutic approaches and the inclusion of a control group, to consolidate the understanding of the efficacy and applicability of RF in the treatment of TN in oncologic patients.

## Conclusions

Our study provided valuable insights into the clinical and sociodemographic characteristics of TN in cancer patients in Mexico. The findings showed a significant reduction in pain following RF treatment, suggesting its potential efficacy and safety in this population. The absence of procedure-related complications further supports its favorable safety profile, making it a promising treatment option.

However, several limitations need to be addressed. The lack of a direct comparison with other treatment modalities and the absence of a control group limit the generalizability of our results. Additionally, the subjective assessment of patient satisfaction was not consistently performed, leaving a gap in understanding treatment outcomes.

Despite these limitations, the study suggests that percutaneous RF is a promising therapeutic approach for alleviating TN pain in cancer patients. Future research should focus on prospective, longitudinal studies with control groups, standardized procedures, and comprehensive patient satisfaction assessments to strengthen the evidence base and optimize pain management in this population. Overall, our study contributes to the understanding of RF treatment for TN in oncology, and further research is needed to improve clinical care for this condition.
